# Inflammatory mechanisms and intervention strategies for sepsis‐induced myocardial dysfunction

**DOI:** 10.1002/iid3.860

**Published:** 2023-05-16

**Authors:** Yuxin Nong, Xuebiao Wei, Danqing Yu

**Affiliations:** ^1^ Department of Cardiology, Guangdong Provincial Key Laboratory of Coronary Heart Disease Prevention, Guangdong Cardiovascular Institute, Guangdong Provincial People's Hospital Guangdong Academy of Medical Sciences Guangzhou China; ^2^ Department of Geriatric Intensive Medicine, Guangdong Provincial Geriatrics Institute, Guangdong Provincial People's Hospital Guangdong Academy of Medical Sciences Guangzhou China

**Keywords:** host‐pathogen interactions, inflammation, myocardial dysfunction, sepsis, targeted therapy

## Abstract

Sepsis‐induced myocardial dysfunction (SIMD) is the leading cause of death in patients with sepsis in the intensive care units. The main manifestations of SIMD are systolic and diastolic dysfunctions of the myocardium. Despite our initial understanding of the SIMD over the past three decades, the incidence and mortality of SIMD remain high. This may be attributed to the large degree of heterogeneity among the initiating factors, disease processes, and host states involved in SIMD. Previously, organ dysfunction caused by sepsis was thought to be an impairment brought about by an excessive inflammatory response. However, many recent studies have shown that SIMD is a consequence of a combination of factors shaped by the inflammatory responses between the pathogen and the host. In this article, we review the mechanisms of the inflammatory responses and potential novel therapeutic strategies in SIMD.

## Graphical abstract

The interaction of inflammatory environment promotes the occurrence and development of myocardial injury induced by sepsis.

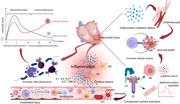

## 1 INTRODUCTION

According to the latest international consensus, sepsis is defined as a life‐threatening organ dysfunction caused by a dysregulated host response to infection, and septic shock is classified as a subtype of sepsis.^1^ Sepsis is shaped by “a combination of factors between the pathogen and the host,” and the guidelines for the management of sepsis and septic shock further highlight the role of organ dysfunction in sepsis.^2^ Notaby, sepsos is a group of syndromes rather than a single desiase. Sepsis‐induced myocardial dysfunction (SIMD), one of the manifestations of organ dysfunction in sepsis, is the main cause of septic shock, and is characterized by myocardial systolic and diastolic dysfunctions.^3^ It has been reported that the mortality rates of patients with septic shock can be as high as 38%.^4^

However, the current knowledge about SIMD is still lacking in many aspects, as reflected in the controversies abounding its definition, identification, and therapeutic management. For example, it is still unclear whether myocardial dysfunction extends from the left to the right ventricle (RV) nd what role diastolic dysfunction plays in SIMD.^5^, ^6^ Additionally, compared to systolic dysfuntion, diastolic dysfuntion is often ignored. Moreover, even when the myocardial decreases, the ejection fraction (EF) of the myocardium remains preserved because the reduced ejection. In terms of treatment, there are no standardized or uniformly specified targeted measures. In the past 30 years, despite the large number of mechanistic studies on sepsis, there has been no satisfactory clinical transformation. The main reasons for these contradictions are the differences in the pathogenic infections and the host response mechanisms in patients. Effective treatment and management need to be adapted according to the evolving disease processes in the patients.^7^

Persistent and excessive inflammation can trigger an unrecoverable inflammatory imbalance in the body, which ultimately leads to tissue and organ damage.^8^ SIMD is unique considering the damage to the other tissues and organs during sepsis. In addition to the direct effects of the pathogen, the host's inflammatory response to the actions of the infectious agents (e.g., activation of the immune cells and the massive release of inflammatory mediators) can also damage the myocardium. Sometimes, these conditions may not be sequential but may act synergistically to amplify the damage to the heart and can be more likely to cause fatal septic shock. These conditions create a vicious cycle and ultimately exacerbate sepsis‐induced damage. This review focuses on the mechanisms of inflammatory response in SIMD.

## 2 MAIN BODY

### 2.1 Immune cell activation in sepsis

**Figure 1:**
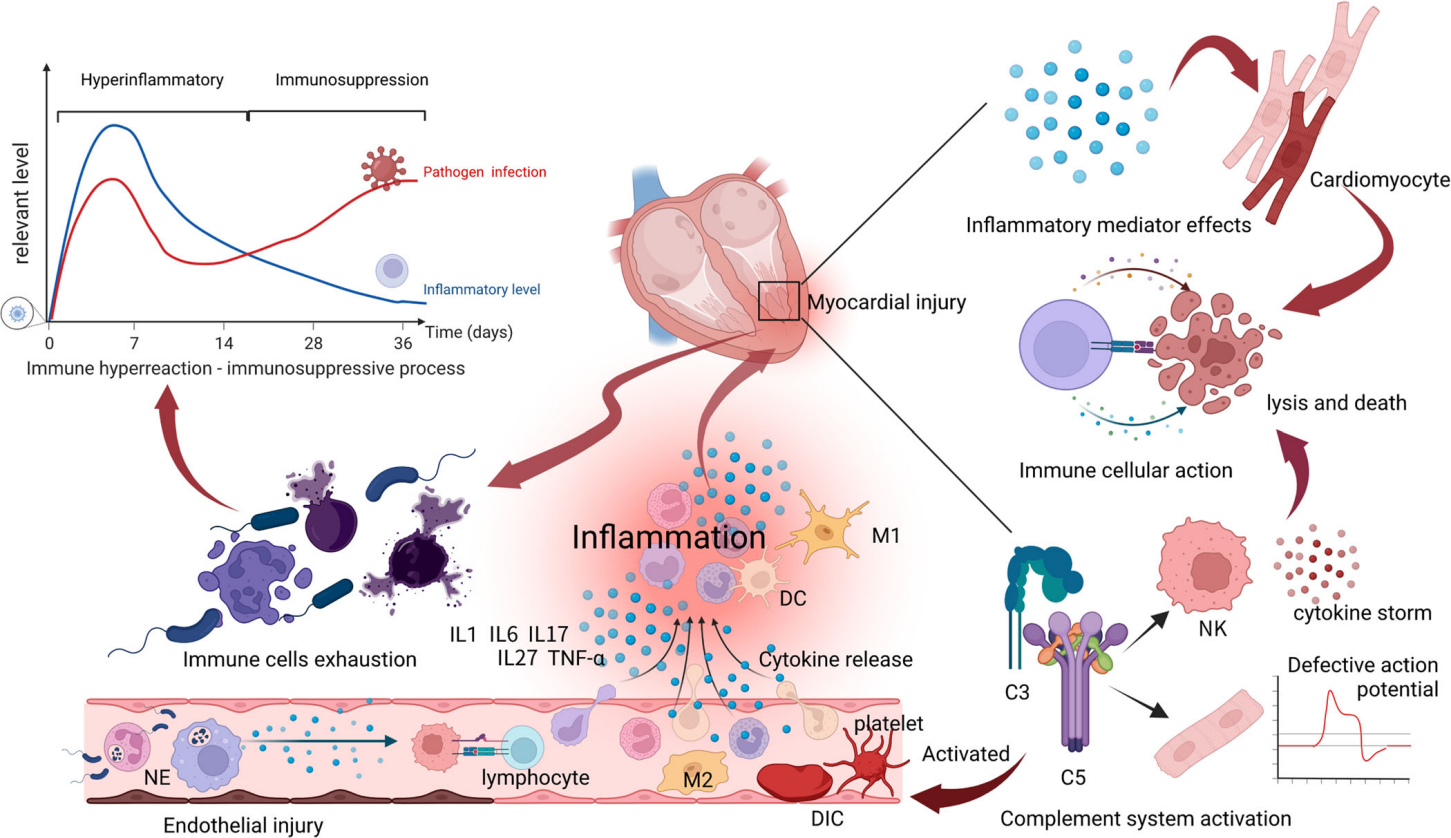
Immune response mediates sepsis‐induced myocardial dysfunction. Early microorganisms activate the immune cells to release immune mediators. Release of inflammatory mediators that target cardiomyocytes leads to cell death. Simultaneously, complement activation amplifies the inflammatory response and affects the myocardial action potential and coagulation system. After several days of infection, the body begins to enter the phase of immune suppression (immune cell failure), and the level of pathogen infection rises again. DC, dendritic cell; IL, interleukin; NE, neutrophil; NK, natural killer cell; M1/2, macrophage 1/2.

Activation of the immune cells is a prerequisite for inflammation. During the occurrence and development of SIMD, the local environment of the myocardium is closely connected with the state of the host, and the two affect each other. Inflammatory mechanisms include proinflammatory and anti‐inflammatory imbalance and immunosuppression. The activation of the host's immune cells after infection forms the basis of the inflammatory response in sepsis. In the interaction between the host and the pathogens, the immune response by the immune cells forms an important cause of SIMD. Therefore, an understanding of the pathogenesis, the exact mechanism behind the inflammatory reactions, and the resulting myocardial injury are the basic premise for developing effective interventions (Figure 1).

One of the most critical routes for activating immune cells is the recognition of pathogen‐associated molecular patterns (PAMPs). PAMPs are a group of highly conserved structures within the cell or in the cell walls of certain pathogens. These structures include lipids, glycoproteins, and nucleic acid components on microbial membranes. They activate immune cells through pattern recognition receptors (PRRs).^9^ PRRs include NOD‐like receptors (NLRs), c‐type lectins (CLRs), and RIG‐I‐like receptors (RLRs); Toll‐like receptors (TLRs) and NLRs are the commonly activated receptors in sepsis.^10^ Damage‐associated molecular patterns (DAMPs) are another group of molecules common to septic immune cells, and these include high‐mobility group box 1 (HMGB1), extracellular cold‐inducible RNA‐binding protein (eCIRP), adenosine triphosphate (ATP), and histones.^11^, ^12^ Chromatin‐associated molecular patterns and metabolism‐associated molecular patterns have also been proposed recently, in succession. The former mainly include DNA, cell‐free RNAs, microRNAs, extracellular traps (ETs), and RNA‐or DNA‐binding proteins. The latter mainly refer to metabolism‐related products: free fatty acids, glucose, advanced glycation end products, cholesterol, oxidized phospholipids, ceramides, and uric acid. These concepts broaden the previous understanding of inflammatory activation in sepsis and demonstrate the complexity of the inflammatory environment in which sepsis occurs.^13^, ^14^ The hosts may exhibit different immune characteristics to different pathogens.^15^, ^16^ Different types of immune cells are activated in different ways, at different times, and play different roles. Neutrophils are one of the most abundant leukocytes in the peripheral circulation, and play a key role in the early recognition of pathogens and initiation of host resistance to infection.^17^, ^18^ Bacterial lipopolysaccharides (LPS) and peptidoglycans, as well as the inflammatory factors (such as chemokines and interleukins) released after tissue injury are important signals for neutrophil activation.^19^ The activated neutrophils are recruited at the site of infection to trap and kill the bacteria by forming neutrophil extracellular traps. They also secrete proinflammatory factors, such as chemokines, growth factors and interleukins, to activate other cells.^20^, ^21^ Monocytes/macrophages are another group of immune cells in an infection. Among them, macrophage polarization (M1/M2) plays a key role that involves a complex regulatory network.^22^, ^23^, ^24^ Such polarization can be initiated not only by external infectious factors, but also by endogenous signaling molecules and pathways, thus maintaining a relative balance.^25^

Similar to neutrophils, dendritic cells (DCs) are also important immune cells, activated in response to sepsis, and involved mainly in antigen presentation and in enhancing immune response lethality.^26^ In the early stages of infection, the host mobilizes its defense system to clear the invading pathogens, a process accomplished by innate immunity. Antigen‐presenting cells (APCs) in the blood, mainly monocytes/macrophages, DCs, and B lymphocytes, are the most critical immune cells activated in the early stages of sepsis. After recognizing the pathogens, the APCs activate, phagocytose, and remove the pathogenic factors and then present the antigen information to T lymphocytes and natural killer cells (NK cells), thereby, inducing their differentiation into corresponding effector cells to enhance and amplify the immune clearance effect. In this process, the phenotype of the immune cells changes constantly according to the function, accompanied by the release of many inflammatory cytokines.^27^, ^28^

### 2.2 Activated immune cells and cytokines damage the heart

Normally, there are different immune cell subsets in the heart, including macrophages, monocytes, lymphocytes, and DCs. These immune cells, together with the cardiomyocytes, form the myocardial immune cluster that regulates the myocardial function. In sepsis, crosstalk between immune cells and cardiomyocytes is common. Neutrophil infiltration is one of the causes of organ damage, including the heart.^29^ Emigrated neutrophils can affect the Na^+^ and K^+^ currents of cardiomyocytes, and then change the rhythm of the myocardium.^30^ In SIMD, macrophage migration inhibitory factor (MIF) mediates interactions between cardiomyocytes and immune cells. It has been shown that inhibiting MIF reduces infiltration of myocardial macrophages in mice, decreases endoplasmic reticulum stress, and alleviates cardiomyocyte diastolic disorders and apoptosis.^31^, ^32^ Previous studies have shown that regulatory T lymphocytes play a reparative role in myocardial tissue after myocardial infarction,^33^, ^34^ but the activation of CD4^+^ T‐cell receptors in the presence of stress can promote cardiac dysfunction.^35^ Whether these immune cells play similar roles in SIMD, remains unknown.

However, in contrast to the direct damaging effects of the immune cells, the cytokines (including those secreted by the immune cells and the tissues) are the cause of the myocardial inflammatory response. Interleukins (ILs) are one of the most common cytokines in the inflammatory response.^36^ As a “strong responsive soldier” of inflammation, IL6 can exacerbate meningococcal septicemia‐induced myocardial depression by targeting P38 signaling in the mitogen‐activated protein kinase (MAPK) pathway.^37^, ^38^ However, a study has shown that moderate concentrations of IL6 may play a protective role in early LPs‐induced sepsis in mice by upregating the nuclear factor erythroid 2‐associated factor 2 (Nrf2) to reduce intracellular oxidative stress levels.^39^ These complicate the role of ILs in regulating myocardial injury in sepsis. Recently, many rare cytokines and new regulatory mechanisms have been studied; these cytokines are not only secreted by immune cells, such as monocytes and macrophages, but also synthesized and secreted by damaged tissue cells, including cardiomyocytes, endothelial cells and fibroblasts^40^, ^41^

### 2.3 Activation of the complement system

In addition to the antibody and cellular responses, complement is another immune response pathway that recognizes common structures on the surface of pathogens, such as bacteria and fungi. The complement system may act through any of the three pathways: the classic, the bypass, and the lectin pathway, all leading to the cleavage of C3 convertase into C3a and C3b to initiate downstream terminal reactions.^42^ In vivo experiments in mice have shown that the complement system of cardiomyocytes can be activated at an early stage.^43^ C5a can synergistically activate NK and NKT cells to mediate the development of a cytokine storm in septic mice, drive the recruitment of NKT and NK cells to the site of infection, and promote the release of TNF‐γ from NK and DC cells.^44^ C5a not only promotes the expression of the reactive oxygen species/NOD‐like receptor protein 3 (ROS/NLRP3) pathway and leads to pyroptosis, but also affects the ion channels and intracellular calcium flux in the cytosol, thereby inhibiting cellular contractility and diastolic functions.^45^, ^46^ Furthermore, C5a receptors on cardiomyocytes can directly affect the physiological function of the myocardium. A previous study in mice, using the patch clamp recording technique, revealed that C5a can affect Na^+^/K^+^‐ATPase, sarcoplasmic/endoplasmic reticulum calcium ATPase 2, and Na^+^/Ca^2+^ exchanger activities, resulting in impaired Ca^2+^ clearance in cardiomyocytes. This impairment leads to defective action potential in myocytes and ultimately affects myocardial contractility and diastolic capacity.^47^

Another important mechanism by which complement affects septic shock is by affecting the function of the coagulation system, which in turn promotes the development of disseminated intravascular coagulation (DIC) and circulatory system disorders.^48^ The presence of DIC can promote the development of multiple organ dysfunction syndrome and greatly increase the risk of patient death.^49^, ^50^ During sepsis, cardiomyocytes from mice with cecal‐ligation and puncture‐induced septicemia release complement‐dependent components, which are characterized by elevated expression of C5a and C5a receptors (C5aR and C5L2).^51^ Activated C3a and C5a can induce platelet activation, and blocking C5a can prevent endothelial cell activation and inhibit platelet function to prevent coagulation.^52^, ^53^

### 2.4 Immunosuppression

Immunosuppression is another feature of immune imbalance in sepsis, which manifests as immune cell anergy (CD4^+^ T lymphocytes, CD8^+^ T lymphocytes, B lymphocytes, and DC exhaustion) and a decreased ability to fight primary bacterial infections; this condition usually develops a few days after infection due to a highly inflammatory response that may cause further secondary infections and worsen disease progression.^54^ It is worth noting that the states of immunosuppression and immune overactivation are not strictly differentiated but show a dynamic range, depending on the type of pathogen, virulence, and defense capacity of the host.^55^ In fact, immunosuppression, following an immune overreaction, may have more serious consequences than the immune response itself.^56^ The exact mechanism of immunosuppression is still unclear, and immune cell apoptosis, endotoxin tolerance, central neuromodulation, reprogramming of the inflammatory response, and metabolic reprogramming are all important contributors to the development of immunosuppression.^57^

One of the main causes of immunosuppression is the inability of immune cells to function, which makes the infection persistent and recurrent. In sepsis, different immune cells have different mechanisms associated with apoptosis and crosstalk, thus differing in their effects on host organs.^58^, ^59^ Chen et al. found that peripheral blood mononuclear cells from sepsis patients had defective energy metabolism, and impaired glycolysis, oxidative phosphorylation, and β‐oxidation, which caused energy failure in the mitochondria and contributed to the loss of immune function.^60^ This conclusion was a significant point of view, and these experiments provided a rational connection between two important mechanisms (mitochondrial disorders and immunosuppression) in sepsis.

During immunosuppression, the inflammatory overreaction in the organ may be transiently relieved to some extent, but this may be followed by fatal exacerbation of the infection. It has been reported that the spleen and lungs can be immunosuppressed during sepsis (e.g., by upregulating the expression of inhibitory receptors on the surface of T cells that infiltrate the organs), thereby worsening the patient's condition.^55^ Apoptotic immune cells can also promote tissue damage in the organs they infiltrate. Immunosuppression has started to gain attention in recent years, and therapeutic strategies have been developed to address this phenomenon. However, because of the organ‐specific nature of these infiltrating immune cells, the exact mechanism of immunosuppression in SIMD is unknown.^61^

### 2.5 Mitochondrial energy metabolism disorders

**Figure 2:**
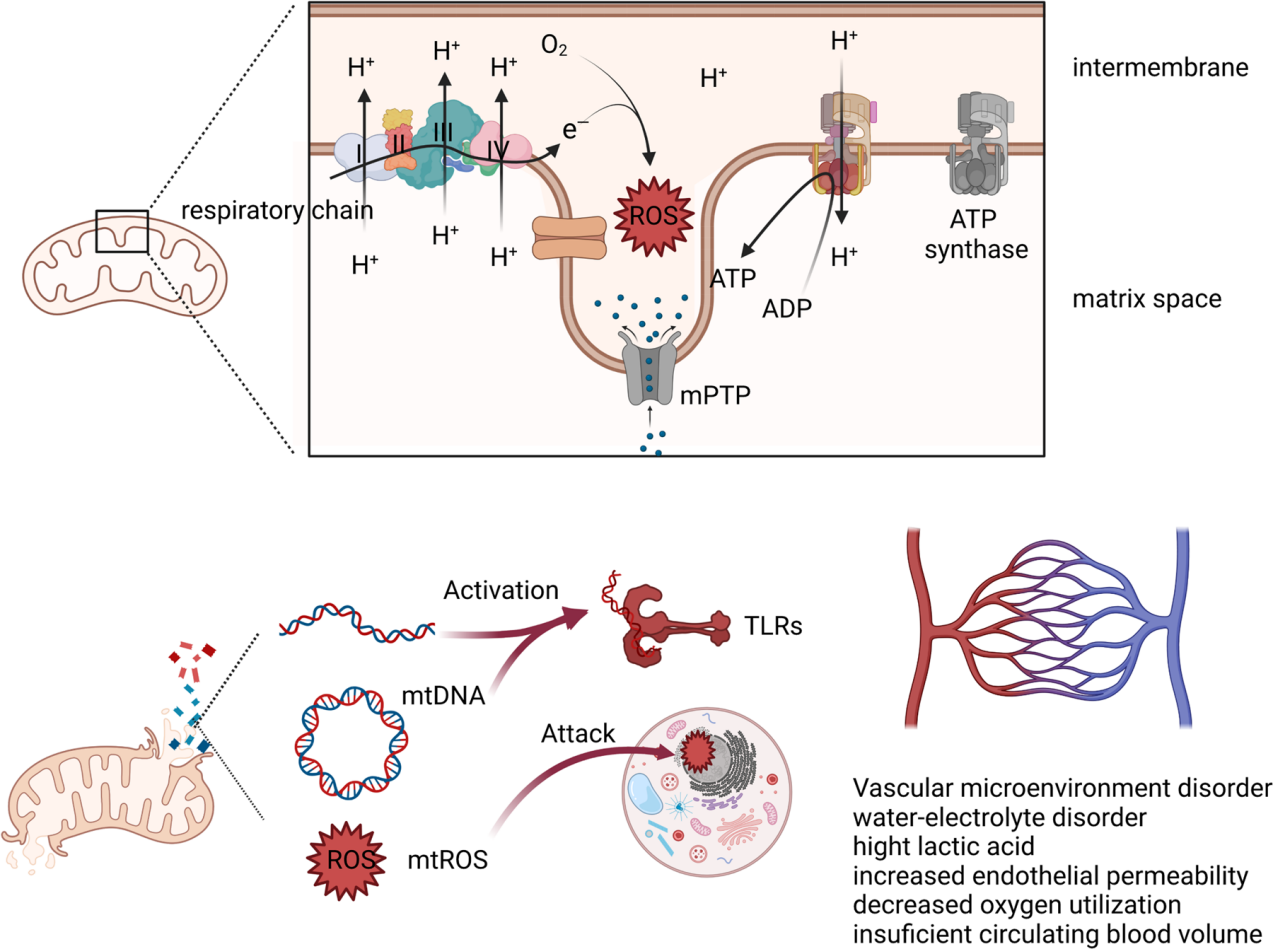
Mechanisms of mitochondrial dysfunction and local metabolic abnormalities. Under the action of inflammatory factors, electrons escape to form ROS, leading to a decrease in ATP production. ROS further attack nucleic acid structures and cause cell damage. The release of large amounts of mtDNA also activates inflammatory pathways. When aerobic respiration is impaired, the local glycolytic pathway gets switched to lactate metabolism, resulting in local metabolic and vasoconstriction disorders that affect the hemodynamic stability. mPTP, mitochondrial permeability transition pore; mtROS, mitochondrial reactive oxygen species; TLRs, toll‐like receptors.

The heart is one of the organs with a high energy requirement, thus it contains a lot of mitochondria, whose energy metabolism is susceptible to injury. These mitochondria can control myocardial organ damage through a variety of quality control mechanisms. (Figure 2).^62^, ^63^ Mitochondrial danger‐associated molecular patterns (mtDAMPs), released after mitochondrial damage and including mitochondrial DNA (mtDNA), mROS, cardiolipin, ATP, and N‐formyl peptide, can activate innate immune receptors to promote inflammatory responses.^64^ mtDNA is recognized by various PRRs and interacts with NLRP3 inflammasomes to promote the maturation of IL1β and IL18.^65^ In a previous study, rats injected with liver mitochondrial DAMPs developed systemic inflammation and acute lung damage, suggesting that circulating mtDAMPs can harm other organs.^66^

Mitochondria are the main source of ROS in cardiomyocytes during sepsis, which is due to the disruption of the electron respiratory transport chain caused by abnormal mitochondrial membrane potential levels and uncoupling of energy transport.^67^, ^68^ Mitochondrial permeability transition is a typical characteristic of mitochondrial damage in SIMD, which is caused by the abnormal opening of the mitochondrial permeability transition pore (mPTP) in the mitochondrial membrane for a long time.^69^, ^70^ Calcium overload, changes in mitochondrial enzymatic activities, decreased ATP production, and mtDNA produced after ROS attack on mitochondria during sepsis can aggravate the energy metabolism disorder of the mitochondria.^71^, ^72^, ^73^ Furthermore, ROS attack normal intracellular structures and disrupt intracellular metabolic homeostasis, and a decrease in ATP accelerates cellular dysfunction, including a decrease in the resistance to scavenging oxides.^74^ Previous studies have shown that LPS‐induced excess ROS can exacerbate hyperglycemia and hypoxia/reoxygenation‐induced myocardial injury by mediating cardiomyocyte death through the NLRP3 inflammasome.^75^ Excessive ROS can also induce HIFα nuclear translocation and downstream events by inhibiting prolyl hydroxylase‐mediated ubiquitination of HIFα, which mediates a shift in energy production from oxygen‐dependent oxidative phosphorylation to hypoxic glycolysis.^76^, ^77^ Under hypoxic conditions, the myocardium can induce HIF1α expression to modulate myocardial glucose uptake and alter myocardial contractility.^78^

Notably though, the above‐mentioned processes should not be considered as independent events, rather as closely interacting elements. Organ damage control during sepsis may also be regulated by mitochondrial fission/fusion events and autophagy events. It has been demonstrated that in animals lacking DNA‐dependent protein kinase, sepsis results in less cardiac damage.^79^ This is a serine/threonine protein kinase that induces mitochondrial fragmentation in kidney injury by interacting with the mitochondrial fission 1 protein.^80^ Additionally, autophagy is essential for the mitochondrial control of SIMD. Activation of mitochondrial autophagy related genes can reduce myocardial damage during sepsis and maintain mitochondrial functional metabolism in mice.^81^ Early inflammatory signals may affect the enzyme metabolism in mitochondria, leading to the production of free radicals. On the one hand, free radicals destroy the potential on the mitochondrial membrane, thereby amplifying the damage to the mitochondria, and on the other hand, they induce the generation of mtDNA, which in turn acts as an intracellular DAMP and aggravates the inflammatory response of cells, thus forming a vicious cycle. Oxidative phosphoric acid uncoupling of mitochondria is a very attractive research direction in these studies. In mammalian mitochondria, the respiratory chain pumps protons into the membrane space, where they produce ATP by pushing ATP synthase back into the matrix. However, when protons do not pass through ATP synthase, they flow into the mitochondrial matrix through some “shortcut paths” in the inner mitochondrial membrane to form “proton leakage.” Te energy utilization efficiency of the cell is reduced, and uncoupling protein (UCP) acts as the “shortcut path.”^82^ UCP2 is one of the UCP family of proteins closely associated with metabolic diseases, such as diabetes, cardiovascular diseases, obesity, and cancer.^83^ Previous studies have identified the upregulation of UCP2 protein in response to LPS‐induced myocardial injury.^84^, ^85^ It is noteworthy that most of the studies suggest that UCP2 plays a protective role in SIMD, which seems to contradict the conventional wisdom. The mechanism behind these phenomena may be explained by the presence of UCPs that make the electrons in the membrane space leak back into the matrix, thus reducing the excessive production of ROS and subsequently reduce the oxidative stress and apoptosis of cardiomyocytes.^86^, ^87^, ^88^, ^89^, ^90^ However, it is unclear whether other members of the UCP play a similar role in SIMD.

### 2.6 Microcirculatory dysfunction

Microcirculatory dysfunction is an important core in the progression of sepsis. Coagulation dysfunction and DIC are the pathophysiological manifestations of sepsis, which may be caused by an imbalance between the coagulant and the anticoagulant systems after activation of inflammatory factors.^91^ The mechanisms by which inflammatory factors contribute to sepsis‐associated coagulopathy have been previously described in detail, including microbial diffusion, neutrophil activation, and DAMP interaction with coagulation factors.^92^, ^93^, ^94^ However, the mechanism underlying the interaction between coagulation and myocardial dysfunction is not well understood. Abnormal activation of the coagulation system may lead to local blood hypoperfusion and abnormal distribution of the peripheral circulation, especially capillary microcirculation disorders.^95^, ^96^

In the early stages, tissue hypoxia caused by hypoperfusion hypoxia may not be obvious because the heart is in a hyperdynamic state and can compensate for the circulation. However, with the peripheral vasodilatation disorders, increased endothelial vascular permeability results in tissue edema and hypovolemia. The circulatory hemodynamics, then, become unstable, perfusion changes from “High Power” to “High Resistance” and further aggravates the dysfunction of the local coagulation system. On the one hand, the formation of microthrombi causes abnormal distribution of peripheral circulation, and on the other hand, it causes local hypoxia in peripheral tissues and accelerates the inflammatory reaction.^97^, ^98^, ^99^ Abnormal blood flow in the local tissues, in turn, promotes local metabolic abnormalities, such as the accumulation of harmful metabolites. Vascular endothelial cells, macrophages, neutrophils, and histiocytes promote the synthesis of excessive nitric oxide (NO) by NO synthase (NOS) under the microenvironmental changes, such as an increase in the inflammatory factors and hemodynamic changes, thus leading to the dysfunction of immune cells, such as the induction of T‐cell apoptosis.^100^ Furthermore, excessive NO can also affect vascular reactivity through downstream effector molecules, such as guanylate cyclase and potassium ion channels, leading to vascular paralysis and further aggravating abnormal blood perfusion in tissues.^101^, ^102^

### 2.7 Autonomic nerve activation

During sepsis, the autonomic nervous system, including the sympathetic and parasympathetic nerves, is abnormally activated, releasing large amounts of catecholamines (mainly epinephrine and norepinephrine) and acetylcholine, which constrict blood vessels and increase peripheral resistance, while enhancing tissue energy metabolism. In addition to the high levels of inflammatory factors, this activation also comes from the abnormal distribution of peripheral blood volume, leading to hypoperfusion and reduced peripheral vascular tension.^103^, ^104^, ^105^ Excessive catecholamine hormones can increase the energy consumption of cardiomyocytes, increase the metabolic burden of myocardial microcirculation, enhance the oxidative stress levels of cardiomyocytes, and affect the myocardial rhythm. The compensatory work of the heart and inadequate coronary blood perfusion accelerate the imbalance in myocardial oxygen supply and demand.^105^, ^106^, ^107^, ^108^

### 2.8 Pathogenic microorganisms mediate damage

In addition to mediating myocardial injury by inducing host immune responses, pathogenic microorganisms can also act directly on cardiomyocytes.^109^, ^110^, ^111^, ^112^ Most microorganisms that can cause sepsis, such as bacteria, fungi, parasites, and viruses (Table 1), can infect the heart. The most common pathogens that cause sepsis are gram‐negative bacteria, including *Escherichia coli*, *Klebsiella pneumoniae*, *Pseudomonas aeruginosa*, and *Streptococcus pneumoniae*, and these pathogens usually spread from peritonitis or pneumonia.^126^

**Table 1:** Common pathogens and mechanism of myocardial injury in sepsis.

Pathogens	Common mechanisms	References
Bacteria		
● *Escherichia coli* ● *Staphylococcus aureus* ● *Streptococcus pneumoniae* ● *Klebsiella pneumoniae* ● *Pseudomonas aeruginosa*	● Bacterial cell wall contains peptidoglycan‐embedded ligands that activate intracellular NFκB signaling through TLR on host cardiomyocyte membranes, increasing the release of inflammatory factors such as TNF‐α, IL‐1β, IL6, and IL8 ● Mediate cardiac inhibition by altering the concentration of calcium ions in the host cell and affecting the contractility of the myocardium ● Disrupt cellular mitochondrial function and structural integrity	[113, 114, 115, 116, 117, 118]
Fungi		
● Candida	● Activate immune cells and the release of inflammatory factors ● Impair endothelial function in coronary arteries	[119, 120, 121]
Virus		
● Herpes simplex virus ● Enterovirus ● Coxsackie virus ● Parvovirus ● Influenza virus	● Recognize and enter cells via ACE2 receptors ● Activate cellular immune responses via TLR and RLR signaling pathways ● Damage the endothelium to impair cardiac microcirculation and induce barrier dysfunction	[122, 123, 124, 125]

Endotoxin, a characteristic cell wall component of gram‐negative bacteria, was previously used to study sepsis. Activation of toll‐like receptor 4 (TLR‐4) is an important mechanism of endotoxin. TLR‐4 promotes the activation of interferon (IFN) regulatory factors, nuclear factor‐κB and MAPK signaling through early myeloid differentiation factor 88 (MyD88)‐dependent and MyD88‐independent pathways and promotes the production of inflammatory factors such as TNF‐α, IL‐6, IL‐8, TNF‐α and granulocyte colony‐stimulating factor.^127^

Similarly, lipoteichoic acid, another conserved bacterial structure (gram‐negative) that mediates septic injury, triggers immune inflammatory damage to the myocardium by activating TLR‐2, TLR‐1, and TLR‐6, which are considered receptor sites for this response.^128^ The most well‐represented gram‐positive bacteria are *Staphylococcus aureus*, which carry toxins with potent pathogenic effects, and some of these bacteria secrete coagulases that predispose tissues to the formation of infectious foci (myocardial abscesses).^129^, ^130^ The misuse of antibiotics in recent years has increased potent virulence factors and made therapeutic interventions more difficult.^131^ The remaining antigenic structures on bacteria can exert cytopathogenic effects, for example, bacterial flagella can mediate innate immune inflammation through TLR5 receptors, leading to acute myocardial contractile dysfunction in rat.^132^

Another common cause of sepsis is viral infection. The mechanism of viral injury in SIMD is very similar to that in viral myocarditis, which sometimes makes it difficult to distinguish between the two; the completion of infection—entry—replication leads to an immune response and cell death.^133^, ^134^, ^135^ Most viruses invade cardiomyocytes by specifically recognizing receptors on the cell membrane. Human immunodeficiency virus, hepatitis C virus, influenza A or B viruses, the coronavirus family, Middle East respiratory syndrome coronavirus, severe acute respiratory syndrome coronavirus (SARS‐CoV), and severe acute respiratory syndrome coronavirus 2 (SARS‐CoV‐2), can recognize angiotensin‐converting enzyme receptor 2 (ACE2) to infect the myocardium and promote cellular damage.^136^, ^137^, ^138^ Pathogens that invade cardiomyocytes can release inflammatory cytokines, such as NF‐α, IL‐1β, and IL‐6 by affecting inflammatory pathways, such as the NFκB, MAPK, and the complement system. These viruses may also change the characteristic phenotypes of cells by affecting intracellular calcium concentrations, mitochondrial conversion, and membrane permeability.^45^, ^127^, ^139^, ^140^, ^141^, ^142^, ^143^

## 3 TREATMENT STRATEGIES

**Figure 3:**
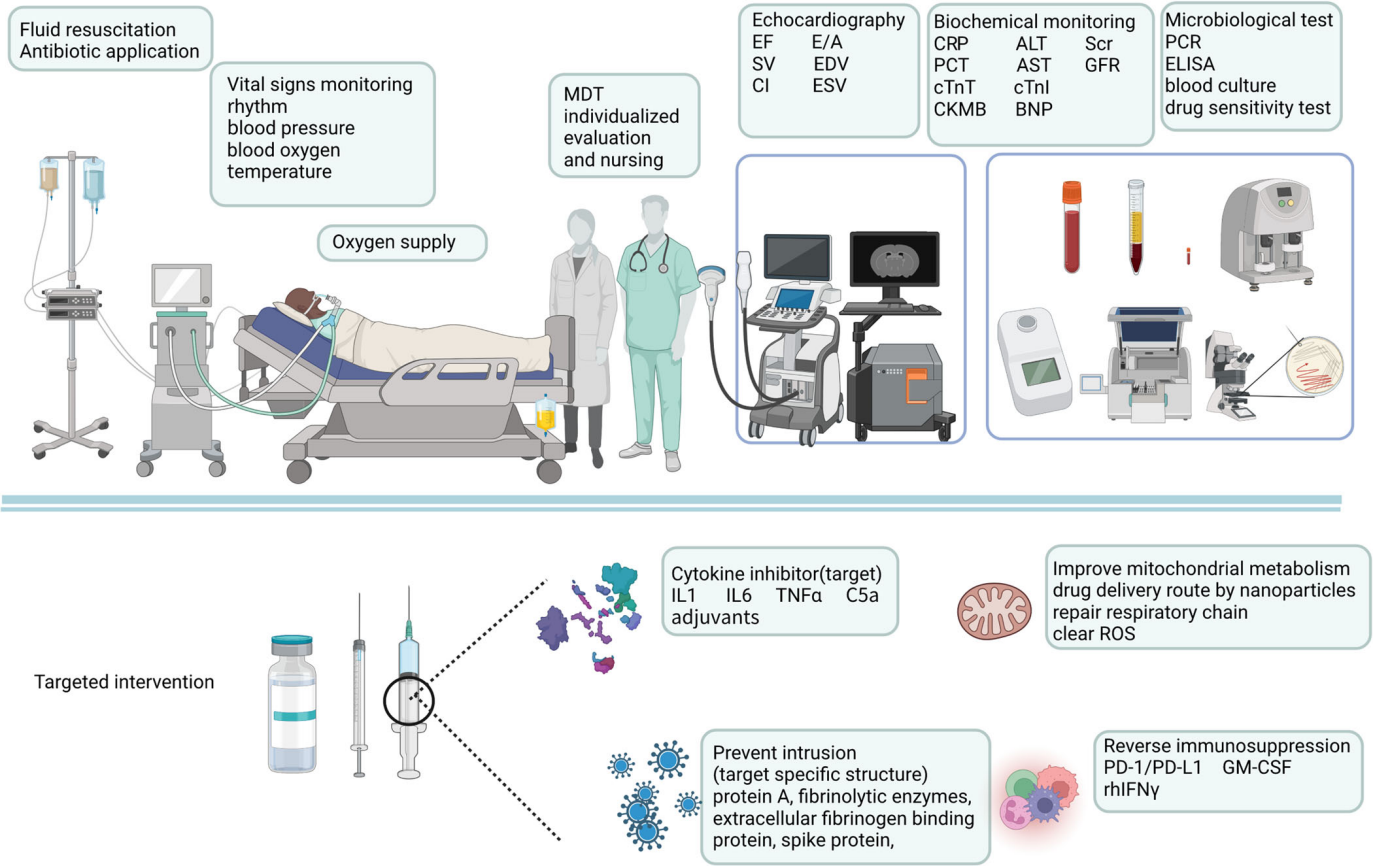
Treatment strategies for septic shock. Based on traditional strategies, a new generation of monitoring and treatment approaches are being developed. Highly sensitive PCR and sequencing technologies allow for faster identification of pathogens. A variety of small molecule targeted drug development is expected to be the future solution. ALT, alanine aminotransferase; AST, aspartate aminotransferase; ELISA, enzyme‐linked immunosorbent assay; GFR, glomerular filtration rate; GM‐CSF, granulocyte‐macrophage colony‐stimulating factor; IL, interleukin; MDT, multidisciplinary treatment; PCR, polymerase chain reaction; rhIFNγ, recombinant human interferon; ROS, reactive oxygen species; Scr, serum creatinine.

The fundamental principles of sepsis treatment and management include early recognition, early control of infection, early restoration of organ tissue perfusion, close monitoring of patient vital signs and markers of organ damage, aggressive fluid resuscitation, active prevention of complications, and optimization of nursing management. The inflammatory reaction to sepsis is systemic, and the prevention and treatment of SIMD should not be limited to the treatment of the heart alone but should be considered from the overall perspective of the host‐pathogen interaction (Figure 3).

### 3.1 Early identification and monitoring

Currently, there are no uniform standards for diagnosing SIMD. The diagnosis and treatment of SIMD depends on a combination of the patient's clinical symptoms and the relevant indicators of cardiac function assessment. However, the early symptoms in patients may not be obvious, especially in severe patients (such as comatose and speech‐impaired), and it is difficult to determine the occurrence of SIMD in a timely manner. Electrocardiography may be a quick and convenient method to evaluate SIMD, however, due to specificity limitations, it cannot be used for the timely determination of SIMD in many cases. Echocardiography (ECHO), especially two‐dimensional ultrasound imaging, is an important means of SIMD diagnosis and monitoring. Routine measures should include left ventricular ejection fraction (LVEF), stroke volume and cardiac index, RV systolic dysfunction, and diastolic dysfunction (E/e’).^144^ However, due to the limitation of consistent dependence, LVEF and other traditional indicators may not fully reflect the degree of myocardial injury. More and more studies support the superiority of speckle tracking ECHO over conventional ECHO in evaluating myocardial function; therefore, this may be the new major monitoring method for SIMD in the future.^145^, ^146^, ^147^

Although they are controversial, biomarkers for SIMD monitoring are still based on traditional common indicators of myocardial damage, such as cardiac troponin T (cTnT) and cardiac troponin I (cTnI), brain natriuretic peptide, and creatine kinase isoenzyme‐MB. Dynamic monitoring of C‐reactive protein, procalcitonin and other inflammation‐related biomarkers are also effective measures to prevent further development of sepsis.^148^, ^149^, ^150^, ^151^, ^152^ In fact, in addition to heart‐related biomarkers, other biomarkers of organ dysfunction should also be included in the category for comprehensive evaluation because damage to other organs will also result in heart damage.^95^

Several novel biomarkers have been developed to identify and track SIMD. Copper (Cu) and zinc (Zn) levels in the blood, Cu/Zn ratio, myeloperoxidase (MPO), pregnancy‐associated plasma protein A (PAPP‐A), and noncoding RNA (Micro) are good performance predictors for SIMD, which can improve the recognition efficiency for SIMD.^153^, ^154^, ^155^, ^156^, ^157^ However, owing to the complex monitoring process and equipment, clinical applications may be limited, and biomarkers should not be used alone.

### 3.2 Targeting the host inflammatory response

The host immune response imbalance has been a major concern in the treatment of sepsis. It should be noted that traditional anti‐inflammatory therapy should be based on the patient's clinical presentation. Precision immunotherapy for sepsis is an advocated research direction, but owing to the complex response mechanisms, current therapeutic advances in this area are still very limited. There are not many directly targeted therapies for inflammation in clinical practice. Most treatments are symptomatic, such as lowering the body temperature, full volume perfusion, and timely removal of pathogens, to reduce the level of inflammation in the patient's body. Clinical practice currently relies heavily on timely infection control, fluid resuscitation, and oxygen administration to achieve this goal. In the Sepsis Save Campaign, patients with sepsis are not recommended to use blood purification techniques such as dialysis to identify potential inflammatory agents unless absolutely necessary. This is because such methods appear to be of little benefit to patients.^2^

Many studies have shown that blocking inflammation‐related pathways can effectively mitigate sepsis‐induced cardiomyocyte pyroptosis, including inhibition of TNF, IL1, IL6, IL7, IL15, coagulation factors, and complement C5a adjuvants.^158^, ^159^, ^160^, ^161^ However, most of these novel molecularly targeted drugs are still in the research and development stage and have not yet entered clinical application. Currently, a number of immunotherapy clinical trials for the treatment of sepsis are underway, such as the application of anakinra (IL‐1 inhibitor), granulocyte‐macrophage stimulating factor, recombinant human INF γ (rhIFNγ), allocetra‐OTS (off‐the‐shelf apoptotic cells) treatments, and Nangibotide (myeloid triggering receptor 1 receptor competitive inhibitor).^162^, ^163^, ^164^, ^165^, ^166^ However, many problems still remain associated with immunotherapy. The inflammatory response is highly heterogeneous across individuals and during different stages. Pure immunotherapeutic interventions may lead to serious consequences. Besides, most current animal models of immunosuppression in sepsis are not very well developed and differ from the actual in vivo environment.^167^, ^168^ Therefore, more in‐depth studies are required to elucidate these mechanisms.

### 3.3 Improving perfusion and microcirculation disturbance

A timely fluid resuscitation can improve the hypoxic condition of local tissues, especially the microcirculation. Currently, norepinephrine, epinephrine, dopamine, and dobutamine are the most commonly used drugs in the clinical treatment of septic shock. These drugs can increase blood pressure and cardiac output in shock patients, and maintain tissue blood flow. The Surviving Sepsis Campaign‐2021 suggests that patients with septic shock should be resuscitated immediately, and recommends the use of norepinephrine instead of dopamine or epinephrine as vasoactive first‐line drugs.^2^ Appropriate use of vasoactive drugs such as amrinone, milrinone, enoxidone and levosimendan on the basis of maintaining blood pressure can help improve the vascular resistance and blood flow status of patients. These have been clinically proven to be effective in the treatment of SIMD. However, the use of these drugs is governed strictly by the patient's organ function status, underlying disease, and progression, which may influence the selection of drugs. As much as possible, the clinician should make a comprehensive assessment of the patient's hemodynamics. Inappropriate vasoactive drug administration and oxygen supplementation may exacerbate the damage. In the absence of appropriate monitoring and care, overly aggressive resuscitation can increase the risk of patient death.^169^, ^170^, ^171^, ^172^ Resurrection fluids should use crystals rather than colloids, as the latter seem to increase the risk of organ damage and death.^173^

In addition to improving local metabolism, interventions aimed at improving myocardial function are currently under study. Previous research showed that the β‐blocker esmolol improved 28‐day mortality, controlled ventricular rate, reduced myocardial energy loss, and improved hemodynamics in patients with sepsis after fluid resuscitation.^174^ However, β‐blockers should still be used very cautiously, and their safety should be evaluated periodically on an individual basis, considering that sepsis itself is a complex environment, and that the application of β‐blockers in the case of hemodynamic instability would increase the risk to patients.^175^, ^176^ Other substances that can improve myocardial oxidation levels, such as melatonin, ferrostatin‐1, luteolin, dexmedetomidine, and other novel drugs, improve myocardial injury in vitro, but their efficacy and safety in vivo have not been verified.^177^, ^178^, ^179^, ^180^

Metabolic resuscitation therapy of mitochondria has recently been proposed to reduce oxidative stress, maintain stable mitochondrial function and improve local tissue metabolism by adjusting mitochondrial metabolism and maintaining normal electron transport chain function.^181^ This strategy includes the use of hormonal drugs, reduction in tissue caloric requirements, promotion of the tricarboxylic acid cycle and ATP production, and the administration of antioxidants.^182^, ^183^ Several mitochondria‐targeted delivery systems based on lipophilic cations and nano pathways have been developed. In a previous study, the surface of the targeted peptide Szeto Schiller 31 (ss31) was modified to carry cyclosporine A (CsA) to inhibit mPTP opening. By using the specificity of the interaction between ss31 and cardiolipin, the researchers carried out targeted drug delivery to mitochondria, and compared to using CsA alone, targeted mitochondrial delivery significantly alleviated the myocardial injury induced by hypoxia/reoxygenation.^184^ In addition to delivery systems based on lipophilic cations, temperature‐dependent mitochondrial drug delivery systems, near‐infrared light‐triggered drug delivery vehicles based on chemical photothermal therapy, and amphiphilic cell‐penetrating motifs have also been developed as promising mitochondria‐targeted drug delivery systems. Research on mitochondria‐targeted interventions is being performed for clinical translation, and several clinical trials targeting mitochondria to improve atherosclerosis, cardiomyopathy, diastolic dysfunction, aging, and peripheral vascular disease are currently enrolling patients.^185^, ^186^, ^187^, ^188^, ^189^ It is expected that mitochondria‐targeted therapy will have broad applications in the prevention and treatment of SIMD.

### 3.4 Targeting pathogen invasion

Antibiotics are still the most effective measures for rapid infection control. The Surviving Sepsis Campaign 2021 recommends that adults with septic shock or a high risk of sepsis be administered antibiotics immediately, and unless there are clear contraindications to antibiotic use, antibiotics should be used as early as possible. To ensure coverage of multiple potential pathogens, use broad‐spectrum antibiotics in combination early before the pathogen is identified, such as carbapenems like meropenem and imipenem/cilastatin, or broad‐spectrum penicillin/beta‐lactamase inhibitors.^2^ A retrospective study of 35,000 sepsis patients in an emergency department showed that hourly delays in antibiotic administration were associated with increased in‐hospital mortality.^190^ A large multicenter, open‐label, randomized trial showed that pre‐hospital antibiotic use in ambulances appeared to improve prognostic outcomes for patients with sepsis.^191^ However, it is still worth mentioning that timely and accurate dynamic assessment of sepsis patients is the primary position of rescue, especially to grasp the indication and time of antibiotic use.^192^, ^193^ It is suggested that in any patient with sepsis, the patient's medical history (such as pneumonia, trauma, local infection, and organ injury) should be combined with the biochemical indicators to make a rapid comprehensive assessment and identify the pathogen as soon as possible, if conditions permit. In addition, attending physicians and caregivers should also take into account local bacterial resistance, such as assessing the risk of methicillin‐resistant *S. aureus* (MRSA), candida, and fungal infections, and adjusting antibiotic regimens to cover the appropriate pathogens.

There have been reports of new therapies that have been developed to stop or slow the extent of pathogenic damage, such as targeted nanoparticles with multifunctional antimicrobial effects, monoclonal antibodies targeting pathogen‐specific structures, surface protein A (SasA), fibrinolytic enzymes, extracellular fibrinogen binding protein (a complement inhibitory protein), teichoic acid, and specific spike proteins.^194^, ^195^, ^196^, ^197^, ^198^ However, these new target therapies are based on the identification of the pathogen, so the rapid identification of the pathogen is still the key to preventing the further spread of infection. Microbial monitoring, though, has shown more promising progress, especially through blood cultures and rapid detection polymerase chain reaction (PCR). Early identification of microorganisms is of great significance for clinical medication, as it can quickly control infection, and also prevent further organ failure in sepsis. As the technology improves, a combination of PCR and blood culture can be used to identify infectious pathogens more quickly. Compared to the traditional blood culture followed by nucleic acid extraction for PCR amplification, the specific sequence amplification of clinical samples can be more rapid and convenient.^199^, ^200^, ^201^, ^202^ Molecular diagnostics for pathogens (nucleic acid sequences, and specific protein fragments) can greatly improve the rapid identification of infectious agents and guide antibiotic dosage.^203^, ^204^ However, molecular diagnosis and prediction in sepsis still face many problems and challenges, and the transformaiional application of the study is not very satisfactory.^205^, ^206^, ^207^, ^208^ These novel technologies are still in the early stages of research, and sensitivity and specificity must be further examined in clinical studies with larger samples. The cost of the assay further hinders the use of these technologies in the clinical setting.

## 4 CONCLUSIONS

Despite many important insights into sepsis and septic shock over the past three decades, SIMD still remains a significant cause of death in sepsis patients. Early monitoring and prevention of organ dysfunction are more important than treatment. In the past, the anti‐inflammatory response was the key to treating sepsis, but new perspectives are increasingly focused on the role of immunosuppression in sepsis. SIMD is not just an organ disorder associated with sepsis but a manifestation of systemic damage, and the prevention and treatment of SIMD should include a holistic view of the host. SIMD remains an important challenge for cardiologists, intensivists, researchers, and patients

## AUTHOR CONTRIBUTIONS

Yuxin Nong and Danqing Yu conceived, designed, wrote, and revised the manuscript together. Xuebiao Wei made referential comments on the content of the manuscript. All authors approved the manuscript for publication.

## CONFLICT OF INTEREST STATEMENT

The authors declare no conflict of interest.
